# Maternal Stress and Child Development: The Moderating Role of Interactive Shared Reading

**DOI:** 10.3390/ijerph22060916

**Published:** 2025-06-10

**Authors:** Chrystian R. Kroeff, Juliana R. Bernardi, Clécio H. Da Silva, Nádia C. Valentini, Marcelo Z. Goldani, Denise R. Bandeira

**Affiliations:** 1 Graduate Program in Psychology, Universidade Federal do Rio Grande do Sul (UFRGS), Porto Alegre 90035-003, Brazil; chrystianr@unisinos.br; 2 Graduate Program in Food, Nutrition and Health, Universidade Federal do Rio Grande do Sul (UFRGS), Porto Alegre 90035-903, Brazil; juliana.bernardi@ufrgs.br; 3 Graduate Program in Child and Adolescent Health, Universidade Federal do Rio Grande do Sul (UFRGS), Porto Alegre 90035-903, Brazil; clecio.homrich@ufrgs.br (C.H.D.S.); mgoldani@hcpa.edu.br (M.Z.G.); 4 Graduate Program in Sciences of Human Movement, Universidade Federal do Rio Grande do Sul (UFRGS), Porto Alegre 90690-200, Brazil; nadiacv@esef.ufrgs.br

**Keywords:** stress, mother–child relationships, family relationships, reading

## Abstract

Research suggests that maternal stress is related to aspects of child development. Positive and stimulating interactions, such as shared reading, may act as protective factors, mitigating the negative effects of maternal stress on children’s development and behavior. This cohort study investigated the predictive relationship between maternal stress and children’s milestones and behavioral problems, with maternal interactive style during shared reading as a moderator. A total of 91 mother–child dyads participated. During the shared reading session, conducted in a private room at a research center, each mother and child interacted freely while reading a book, without specific instructions. The sessions were video-recorded and later analyzed by trained researchers using an established method. Children’s data were assessed using questionnaires completed by the mother on the same day. Descriptive, bivariate, and multivariate analyses were performed to build regression models with moderation analysis. Results revealed that maternal stress significantly predicted children’s internalizing and externalizing symptoms. Notably, more affectionate interactions during shared reading can moderate the effect of stress on children’s externalizing difficulties. These findings suggest that fostering positive and engaging interactions between mothers and children, such as shared reading, may have a beneficial impact on children’s behavioral development, even in the presence of maternal stress.

## 1. Introduction

The arrival of a new family member often brings moments of joy and fulfillment, particularly when this event results from careful planning or a strong desire to have children. Even an unplanned pregnancy, however, can be an accomplishment worth celebrating. At the same time, the expectations and anticipations of those caring for the child may lead to feelings of frustration and uncertainty. In short, the transition to parenthood and the welcoming of a newborn is an experience marked by a wide range of emotions, often contradictory in nature [1,2].

Caring for a newborn and young children typically alters daily routines in significant ways. New demands, some of which may be unfamiliar, can increase the workload. Sleep quality and duration often decline, adding to the challenges. The absence of a strong support network during this time can further exacerbate these challenges [3]. These factors, among others, contribute to heightened stress related to parenting demands [4].

Reactive responses of the body—both psychological and physiological—to environmental events perceived as uncertain, threatening, or exhausting can be adequately defined as stress [1]. Specifically in the parenthood context, some researchers have proposed frameworks for what is termed parental stress, which may be maternal or paternal, depending on the focus [5,6]. This stress response is characterized by parents’ reactions to the new challenges imposed by the demands of parenthood [1].

Significant parental stress can have numerous consequences. Studies have shown associations between stress and lower parental self-efficacy [2], as well as increased rates of maternal or paternal depression [5], anxiety [7], and notably, altered parenting practices or parent–child interaction styles [8,9,10]. Beyond its effects on caregivers, parental stress has been related to various aspects of child development, including cognitive, socio-emotional, motor, and linguistic milestones [7,11,12,13].

In one study conducted in Canada, researchers explored how psychosocial variables in mothers with a history of risk predict maternal interaction styles [14]. The study evaluated mother–child dyads, with children aged two to six years, using psychometric scales and observational measures. Interaction style, referred to as “parental guidance” by the authors, was classified as either directive or non-directive. A directive style is characterized by mothers who seek to exert greater control over their child’s behavior, while a non-directive style is more responsive and autonomy-supportive.

In Briscoe et al.’s study [14], results indicated that maternal stress was strongly correlated with directive interaction style. Mothers who experienced higher levels of stress were more likely to adopt controlling behaviors during interactions with their children. In regression models, maternal stress was a statistically significant predictor of this parenting style, even when controlling for factors such as age, socioeconomic status, and maternal education. Similarly, Karam et al. [15] reported that high levels of maternal stress were associated with less positive adult–child interactions.

In a related study, Ionio et al. [7] investigated the influence of paternal stress on maternal interactions with their children. Their study, which focused on Italian families, also examined the potential impacts on the development of preterm infants. As expected, poorer child health conditions were linked to higher parental stress levels. Their findings revealed that in families where fathers scored higher on stress scales, mothers tended to engage in less responsive and more intrusive interactions with their children, focusing less on the baby’s needs and more on their anxieties.

In another study, Winstone et al. [2] examined the predictive influence of parental stress, parental satisfaction, and dyadic interaction dysregulation on maternal and child well-being one year later. The study involved families of Mexican origin living in the United States, with longitudinal assessment conducted when the children were 12, 18, 24, and 36 months old. The results showed a negative association between parental stress and satisfaction across all data points. Furthermore, more stressed mothers tended to exhibit greater dysregulation in dyadic interactions, a factor the authors suggested could be linked to child behavior problems.

Despite the growing body of evidence on this topic, few studies have explored the impact of parental stress specifically within the context of shared reading interactions [16]. Most of the studies so far focus on dyadic exchanges during play, whether structured or unstructured. It is also worth reflecting on the potential positive impact of shared reading interactions, not only in reducing maternal stress but also in moderating the effects of maternal stress on child development.

As highlighted in this work, shared reading can be understood as an opportunity for high-quality interaction. The nature of shared reading—fostering close, joint exchanges of various contents and narratives—makes it particularly valuable. However, this topic remains underexplored in the literature. One Brazilian study addressed this issue [17], investigating how parents engage in shared reading with their children in a natural, non-interfered setting. This study identified key strategies, including organizing the physical space, interacting with the book, storytelling approaches, and the affective relationship between the adult and the child. Most studies [10,18] on this topic, however, have focused on intervention rather than employing other investigative methods, such as the one proposed in the present article.

The discussion also extends to other potential impacts of maternal and paternal psychosocial variables on children. As previously noted, parental stress may indirectly affect child development [12,19]. Karam et al. [15] assessed the impact of parental stress reactions on developmental variables in one-year-old children. Prenatal and postnatal stress (measured approximately two months after the baby’s birth) were examined. It was observed that significant findings indicated that stress was a predictor of lower motor and socio-emotional skill development. This result was consistent when analyzing paternal stress separately.

Given these findings, it is evident that evaluating the potential impacts of parental stress on key aspects of child development—whether maternal, paternal, or related to the primary caregiver—is crucial. Additionally, most studies reviewed report data from developed countries, whose realities are not always analogous to Brazil’s. Notably, Pereira et al. [4] conducted a study investigating possible influences of stress and anxiety among Brazilian mothers on their children’s developmental aspects. The authors found marginal results suggesting potential impacts on motor and language development, but emphasized the need for larger samples in future studies.

Therefore, it is important to establish a clearer understanding of the relationships and predictive power of parental stress measures on how mothers interact with their children [8]. Similarly, understanding how psychosocial variables such as maternal stress influence child characteristics provides empirical support for interventions in this field. Furthermore, when this interaction occurs through shared reading, it should be investigated as a valuable tool for mitigating the effects of elevated parental stress.

Thus, the present study aimed to investigate the predictive relationship between maternal stress and children’s emotional and behavioral characteristics moderated by maternal interactive style during shared reading. The specific objectives were: (i) to investigate the predictive power of maternal stress on children’s emotional and behavioral difficulties, (ii) to examine the predictive power of maternal stress on lower scores in socio-emotional milestones in child development, and (iii) to determine whether variables related to maternal interactive style during shared reading can moderate the effects of maternal stress on children’s emotional and behavioral characteristics.

## 2. Materials and Methods

### 2.1. Participants

The sample consisted of 91 mother–child dyads. The mothers were recruited during their pregnancies from public hospitals in Porto Alegre, RS, specifically the Hospital de Clínicas de Porto Alegre (HCPA) and the Grupo Hospitalar Conceição (GHC). This sample was part of a cohort study tracking 400 children and their mothers from birth through the final data collection point [20,21]. The children were between three and six years old at the time of the data collection.

### 2.2. Procedures

This study utilized data from a cohort research project in collaboration with the Center for Child and Adolescent Studies (NESCA), a research group associated with the Graduate Program in Child and Adolescent Health at the Universidade Federal do Rio Grande do Sul (UFRGS). This research, titled Impact of Perinatal Environmental Variations on Newborn Health (IVAPSA), primarily aimed to investigate potential associations between the intrauterine environment and children’s behavior, metabolism, and neurodevelopment during their early years [20,21].

The project began in 2012, following 400 newborns through several stages: postpartum, seven and fifteen days of life, and at one, three, and six months of age. The present research was conducted in the final phase of the IVAPSA project, re-evaluating participating children aged three to five years. The project has since been completed.

Measures were collected throughout the study, including nutritional, anthropometric, and biological data (such as blood samples). During the final phase, researchers from the Study, Application, and Research Group in Psychological Assessment (GEAPAP), linked to the Graduate Program in Psychology at UFRGS, conducted psychological evaluations. These included assessments of children’s cognitive, socio-emotional, communicative, motor, and adaptive development and observations of mother–child interactions during shared reading and free play sessions.

The data collection for the present study took place in a laboratory setting at the Clinical Research Center (CRC) of HCPA. These sessions involved evaluations of maternal perceived stress levels and interactional contexts. The dyads were recruited via telephone by cohort researchers and attended the CPC on Saturday mornings for the study procedures. Interaction sessions were conducted in a separate room, where the mother and child were alone and recorded on video while engaging in shared book reading and free play.

At the start of the procedure, participants were informed that they would receive three bags: the first contained a children’s book, while the other two—provided after the reading session—contained toys. Trained researchers later analyzed the videos following a predefined method (details in the Section 2.3). Child developmental characteristics—such as developmental milestones and behavioral problems—were assessed using psychometric tools completed by the mother on the same day as the filming. Questionnaires were administered by psychologists or psychology students who were part of the IVAPSA team.

### 2.3. Instruments and Measures

Sociodemographic questionnaires were administered at each data collection point throughout the study. For the present analysis, the following sociodemographic variables were used for sample description and as control variables: maternal age, maternal education (in years), self-identified maternal race, household income (reported in Brazilian reais and as multiples of the minimum wage), number of children, child’s age, child’s sex, and frequency of attendance at early childhood education institutions.

Maternal stress was measured using the 14-item Perceived Stress Scale (PSS) [22], a widely used self-report instrument with a five-point Likert scale (from 0 = “never” to 4 = “always”). The scale includes both positively and negatively worded items. In a Brazilian sample, Faro [23] confirmed its bifactorial structure, reported a Cronbach’s alpha of 0.77, and found evidence of convergent validity.

Maternal interaction style during shared book reading was assessed using an observational system developed by Kroeff et al. [24] that categorizes behaviors into six dimensions: (i) teaching, (ii) prompting, (iii) connecting, (iv) reinforcing, (v) modulating, and (vi) narrating. Two trained observers coded video recordings, calculating behavior frequencies to generate dyad scores. Interrater reliability was satisfactory, with intraclass correlation coefficients ranging from 0.71 to 0.91.

The Strengths and Difficulties Questionnaire (SDQ) is a 25-item scale assessing behavioral problems and prosocial skills in children aged 2–14. It comprises five factors: emotional problems, hyperactivity/inattention, conduct problems, peer relationship problems, and prosocial skills—the only strength-related dimension. Validity studies in Brazilian samples by Woerner et al. [25] and Silva et al. [26] showed significant discriminative power between clinical and non-clinical groups based on parent or teacher reports. Reported reliability included a Cronbach’s alpha of 0.80 and a test-retest correlation of 0.79.

The Dimensional Inventory of Child Development Assessment (IDADI) evaluates developmental milestones in children aged 4–72 months based on parental reports. Developed and validated in Brazil by Silva et al. [27,28], it covers cognitive, motor (gross and fine), communication (receptive and expressive), socio-emotional, and adaptive behavior domains. Each domain includes items corresponding to age-appropriate milestones. The authors demonstrated strong content validity, with inter-domain correlations ranging from 0.87 to 0.95 and high reliability coefficients between 0.97 and 0.99.

### 2.4. Ethical Procedures

All participants provided informed consent by signing a Free and Informed Consent Form. Mothers were informed that their participation was voluntary and that they could withdraw at any time without repercussions or impact on their care at the hospital where they were recruited. Throughout the cohort, participants received feedback on the results of medical and psychological assessments for mothers and children. The project was submitted to and approved by the Ethics Committees of HCPA (CAAE: 65190217.5.0000.5327).

### 2.5. Data Analysis

Preliminary univariate analyses were conducted to examine the distribution of each variable included in the study, analyzing frequencies, means, standard deviations (±*SD*), and normality criteria (using skewness and kurtosis metrics). Internal consistency analyses were also performed for the PSS-14 and SDQ scales using the present study’s sample. Bivariate analyses were conducted to investigate correlations between measures, aiming to evaluate potential multicollinearity among independent variables and the relevance of control variables for the studied outcomes.

Pearson’s linear correlations were used for this purpose. Mean comparison tests (*t*-tests and ANOVA with Tukey post hoc tests for categorical variables with more than two groups) were employed to compare groups according to sociodemographic variables. Finally, multivariate multiple linear regression analyses were used to propose predictive models in which maternal stress (PSS-14) served as the predictor variable alongside maternal interactive style during shared reading. The outcome variables were derived from the SDQ scale, which assessed children’s emotional and behavioral difficulties, and the IDADI instrument, which evaluated dimensions of child development, specifically focusing on socio-emotional and cognitive dimensions for this study.

Furthermore, the interaction between maternal stress and interactive style during shared reading was assessed to identify potential moderating effects of the reading style on the relationship between maternal stress and child characteristics. Data analyses were performed using SPSS v.26, with the PROCESS extension package used to evaluate moderation effects.

## 3. Results

### 3.1. Descriptive Results

The total sample consisted of 91 mother–child dyads. The mothers in this study had a mean age of 32.49 years (*SD* = ±7.64), ranging from 20 to 58 years. Their years of education ranged from 4 to 25, with a mean of 10.50 (*SD* = ±3.47). All participants were literate. Regarding race, 54 mothers self-identified as White (59.3%), 21 as Black (23.1%), 15 as Mixed-race (16.5%), and one as Asian (1.1%). Thus, the sample included 37 self-identified non-White participants (40.7%).

In Brazilian reais, household income ranged from BRL 600 to BRL 28,000 (the latter being an outlier—excluding this case, the highest reported income was BRL 9900). The mean household income was BRL 2499.93 (*SD* = ±3113.53). In terms of minimum wages at the time of data collection, the mean household income was 2.62 (*SD* = ±3.26) minimum wages. Just over half of the sample (51.60%) reported earning up to two minimum wages, 20.90% reported earning between two and three minimum wages, 13.20% earned between three and four minimum wages, and 13.20% reported earning more than four minimum wages.

The number of children per family ranged from one to six, with the vast majority of mothers in this study (81, representing 90%) having no more than three children at the time of data collection. The children’s ages ranged from 36 to 78 months (*M* = 28.88, *SD* = ±10.96) at the time of data collection. Among the children, 56 were female (61.5%). Additionally, most children regularly attended early childhood education institutions, totaling 75.8% of the sample (69 children). Only 22 participants (24.2%) did not attend early childhood education institutions.

Descriptive statistics for the instruments used to assess maternal stress (PSS-14), maternal interactive style during shared reading, children’s emotional and behavioral difficulties (SDQ), and developmental milestones are presented in Table 1. Skewness and kurtosis values were close to 1, with most values below this threshold, indicating good symmetry in score distributions within the evaluated population. The Cronbach’s alpha values for the scales used were satisfactory, consistently above 0.70 and exceeding 0.80 for some scales. Measures of interactive style during shared reading were derived from behavioral observation, and the IDADI scores were calculated based on the standardized scoring system of the published instrument. Therefore, internal consistency measures are not reported for these variables.

### 3.2. Correlation Analyses

Bivariate Pearson’s linear correlation analyses were conducted among the target variables. No significant direct relationships were found between stress measures and the categories of maternal interactive style during shared reading, allowing for the construction of regression models with these two variables as predictors. Regarding the relationship between stress and outcome variables, both PSS-14 factors and the total score showed statistically significant correlations with the SDQ, including internalizing and externalizing factors, as well as the scale’s total score. The PSS-14 also presented a significant association with the IDADI dimension evaluating socio-emotional development.

Among the measures of maternal interactive style during shared reading, the modulation category showed statistically significant correlations with all three SDQ measures (internalizing, externalizing, and total). It was also associated with the IDADI dimension assessing cognitive development. Based on the statistical significance criteria, the other interactive style measures were not significantly associated with any variable in this study. Table 2 details the results of the bivariate analyses for the measures of interest relevant to this study’s objectives.

The relationships between sociodemographic variables and the study’s target measures were analyzed. First, continuous variables related to participants (maternal age, child’s age, absolute income, number of children, and maternal years of education) were examined. Then, groups were compared based on categorical sociodemographic variables (self-reported race, income brackets regarding minimum wages, and the child’s sex).

Maternal age did not show significant correlations with most variables but had weak negative correlations with total PSS scores (*r* = −0.23; *p* < 0.05) and the PSS-14 resources factor (*r* = −0.25; *p* < 0.05). It also showed low Pearson negative correlation coefficients with total SDQ scores (*r* = −0.27; *p* < 0.05) and the externalizing factor of the same scale (*r* = −0.27; *p* < 0.05). Additionally, maternal age was significantly associated with the cognitive development measure of the IDADI (*r* = 0.27; *p* < 0.05). On the other hand, child age showed only one weak significant positive correlation with the internalizing factor of the SDQ (*r* = 0.25; *p* < 0.05).

The number of children in the family showed some significant correlations with small effect sizes, such as total SDQ scores (*r* = 0.22; *p* < 0.05) and the internalizing factor of the SDQ (*r* = 0.21; *p* < 0.05). An exception was found between the number of children and the cognitive dimension of the IDADI, which had a notable effect size (*r* = −0.40; *p* < 0.01).

The most related sociodemographic variable in this study was maternal education. It showed statistically significant correlations with medium effect sizes for all SDQ scores: total (*r* = −0.36; *p* < 0.001), internalizing (*r* = −0.38; *p* < 0.001), and externalizing (*r* = −0.25; *p* < 0.05). Additionally, a significant correlation was found between maternal education and the modulation category in maternal interactive style during shared reading (*r* = 0.32; *p* < 0.01), as well as the cognitive dimension of the IDADI (*r* = 0.31; *p* < 0.05). Household income was also related to modulation during shared reading (*r* = 0.26; *p* < 0.05), which can be better explained by the strong association between income and maternal education (*r* = 0.63; *p* < 0.001).

### 3.3. Group Comparisons

Differences between groups divided by income brackets were analyzed, categorizing mothers into groups earning less than two minimum wages (*n* = 47), between two and three minimum wages (*n* = 19), between three and four minimum wages (*n* = 12), and more than four minimum wages (*n* = 12). This analysis addressed outlier cases of high income compared to the rest of the population. No statistically significant differences were found among these groups for SDQ scores or the socio-emotional dimension of the IDADI. A significant difference was observed only between the highest income bracket and the two lowest brackets when considering the cognitive dimension of the IDADI [*F*(3) = 3.44; *p* < 0.05]. No significant associations were found for the other variables. Lastly, differences between girls (*n* = 56) and boys (*n* = 35) were examined for this study’s variables of interest. No statistically significant discrepancies were found between groups divided by child sex for any variable.

### 3.4. Regression Models

The bivariate analyses guided the construction of multivariate regression models, which evaluated moderation and covariance effects. The models included maternal stress (PSS-14) and maternal interactive style modulation as predictor variables, with maternal education as a covariate due to its relevance in the literature and within this sample. Child outcomes were measured using the SDQ and IDADI.

A post hoc power analysis was conducted using G*Power 3.1 to evaluate the adequacy of the sample size for detecting interaction effects in multiple regression. Assuming a small to medium effect size (*f*^2^ = 0.08), α = 0.05, and four predictors, the achieved statistical power was 0.53. While below the conventional threshold of 0.80, this level of power is acceptable within the exploratory scope of the study. Nonetheless, the limited power to detect moderation effects is acknowledged as a study limitation and reinforces the need for replication with larger samples.

Four models were tested. In Model 1 (SDQ internalizing), maternal stress and maternal education were significant predictors, while modulation and its interaction term were not. Model 2 (SDQ externalizing) also showed significant effects for stress and education; the interaction between modulation and stress approached significance (*p* = 0.07), with a bootstrapped 95% confidence interval ranging from −0.001 to 0.044. Model 3 (SDQ total) mirrored these findings, with a marginal interaction effect (*p* = 0.07) and a bootstrapped confidence interval of −0.003 to 0.070. Finally, in Model 4 (IDADI socio-emotional), maternal stress was again a significant predictor, but modulation, the interaction term, and maternal education were not.

A summary of model statistics is provided in Table 3.

**Figure 1:**
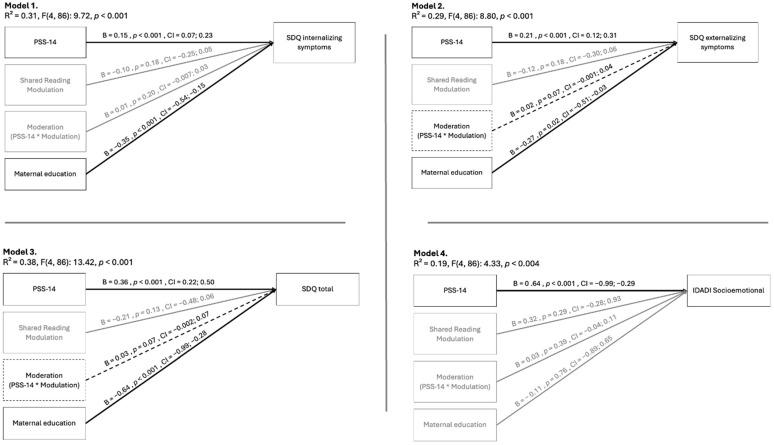
Summary of the four regression models tested.

Figure 1 presents the graphical representation of these models. The conceptual diagrams below summarize the four tested models. Variables in black represent statistically significant predictors, dashed lines indicate borderline effects, and variables in gray were not statistically significant.

## 4. Discussion

The analyses revealed no direct relationship between maternal stress and aspects of the mother’s interactive style during shared reading, suggesting that mothers may tend to regulate their emotional state well when interacting with their children through a children’s book. However, it is expected that higher stress levels in adults will impact the quality of interactions with their children, potentially leading to negative outcomes in child characteristics.

A study in China examined the relationships between parental stress, parenting styles, and perceived behavioral problems in children aged 3 to 7 years [12]. The authors found evidence that higher stress levels predicted increased behavioral difficulties in children. Additionally, the study observed that parental stress was positively correlated with negative parenting styles and that parental interaction styles partially mediated the relationship between stress and child behavior problems. These findings align with the present study’s results. However, this study’s lack of a direct relationship between maternal stress and parenting style during shared reading may be tied to the specific interactive context examined.

Shared reading can provide a positive context for adult–child interaction [29]. Interaction through a children’s book may enhance the quality of exchanges between mother and child by offering rich visual and linguistic content, as well as cognitive and emotional stimulation. This reinforces the hypothesis that book-mediated interaction can help adults regulate their behavior and engage more positively and affectionately during shared reading, even under stress. In other words, shared reading may serve as a protective interactional context that promotes development for both the child and the adult.

On the other hand, maternal stress appeared to be related to child characteristics, including emotional and behavioral difficulties as well as developmental aspects. These relationships may suggest that stressed adults could influence their children’s behavioral and socio-emotional repertoire. Over time, repeated interactions between children and stressed parents may impact developmental outcomes. As previously mentioned, highly stressed mothers and fathers tend to exhibit lower-quality interactions with their children [12,19], using fewer words, showing less affectionate behavior, and adopting more punitive and rigid stances. A study conducted in the Czech Republic [30] investigated a large sample of pregnant women, examining the relationship between prenatal depressive symptoms, anxiety, and psychosocial stress with maternal–fetal attachment. Women with lower levels of prenatal depression reported higher quality attachment to their babies.

Regarding this, Fonseca et al. [19] conducted a study investigating the role of what they term psychological flexibility in promoting more positive parenting styles. The authors examined how more flexible mothers could reduce the impact of their stress levels on their parenting style. They found that lower flexibility was associated with less authoritative parenting while correlating with more authoritarian parenting styles. The study demonstrated how parents with better self-regulation capacities can manage stress more effectively, reducing its impact on their children’s emotional and social development.

Recent findings reinforce the notion that maternal mental health and contextual factors strongly influence mother–infant bonding. A large-scale study by La Rosa et al. [31] demonstrated that postpartum depressive symptoms, sleep disturbances, and a lack of social or partner support were key negative predictors of bonding quality. Conversely, maternal relationship satisfaction and perceived support played a protective role. These findings underscore the importance of future research incorporating contextual variables such as social support. Intervention programs that promote high-quality mother–child interactions may, therefore, play a beneficial role in supporting child development.

Furthermore, another hypothesis may explain the results of the present study. The direct association between maternal stress and impairments in children’s behavioral and socio-emotional development might not fully explain the correlations found in these analyses. This is because the instruments used to assess child characteristics relied on hetero-reports completed by mothers, the same adults experiencing varying stress levels. Thus, it is plausible to consider that stressed mothers do not necessarily have children with greater behavioral and emotional difficulties but rather perceive their children as more challenging or problematic. In this case, the perception of the child’s characteristics could be influenced by the adult’s stress level. In other words, stressed mothers may be more susceptible to motherhood’s challenges and, therefore, describe their children as more difficult. This raises a key interpretive issue: rather than reflecting actual increases in child difficulties, the results may reflect variations in maternal perception influenced by stress.

This issue was explored in a study conducted in Italy [32] with a large sample of mothers and fathers of children aged 2 to 10 years. The authors investigated the relationship between different parenting styles and child difficulties, which were also measured using the SDQ scale. They considered the mediating role of parents’ perceptions of having a “difficult” child, rather than the actual characteristics of the child, which was achieved using a subscale of an instrument measuring parental stress levels, specifically focusing on parents’ perceptions of their child’s self-regulatory abilities. Results showed that parental interpretation partially explained the relationship between parenting styles and child difficulties. However, aside from the influence of parental perception, parenting style still had a significant effect on child variables.

This interpretive layer is essential when considering the use of self- and hetero-report instruments, which are vulnerable to subjective distortions, particularly under conditions of psychological distress. Therefore, the present study’s findings must be interpreted with caution, recognizing that maternal stress may not only influence children’s development but also how that development is perceived and reported. Future research would benefit from including multiple informants (e.g., fathers and teachers) or observational measures to disentangle perceptual biases from behavioral realities.

Among the aspects of maternal interactive style examined, the standout variable was modulation. Modulation refers to how the mother adjusts and varies her tone of voice and emotional prosody during shared reading. Operationally, the degree to which shared reading includes markers of affect and emotional variation. Low modulation, for instance, would indicate a more monotonous, linear, and less theatrical or playful reading style. Modulation during shared reading was associated with child characteristics, particularly emotional and behavioral difficulties (internalizing and externalizing), and showed a modest association with cognitive development.

Prosodic modulation in caregiver–child interactions has been highlighted in the literature as an important variable in child development, especially regarding word learning and language development. For example, Shi et al. [33] investigated modulation in English- and U.S.-based caregivers as they played with children and introduced different toys. The study observed that caregivers used strategies such as slowing their speech, increasing vocal intensity, and raising pitch during specific expressions, often adapting to the child’s linguistic abilities. Notably, the authors found that pitch variation was the primary predictor of immediate word learning and children’s vocabulary size a year later.

Valladares [34] investigated the association between emotional variation in parents’ reading to their children and qualitative aspects of the interaction between parents and children. It also investigated whether parental prosody during shared reading correlates with parental language and children’s reading abilities. The findings demonstrated significant associations—controlling for variables such as parental education and reading skills—between prosodic measures during reading interactions and the quality of parent-child interaction. The author noted the need for further research on the role of prosodic aspects in adult–child shared reading.

The correlations observed in this study suggest that mothers with a more modulated interactive style are associated with children who exhibit fewer emotional and behavioral difficulties, both internalizing and externalizing. This finding aligns with the idea that more affectionate communication, expressed through shared reading interactions, could foster positive outcomes in children’s emotional and behavioral resources. Therefore, in studies and interventions involving shared reading, modulation (or analogous behaviors) may serve as a focal point for promoting development through adult–child interaction and creating high-quality interactive contexts.

At the same time, the explanatory hypothesis previously discussed regarding the effect of maternal stress on child characteristics remains valid here. Specifically, mothers with a higher degree of modulation, expressed through greater affect and emotional variation during shared reading, may perceive or interpret their children as having fewer developmental difficulties. It is worth noting, however, that stress and modulation were not related in this study’s findings, indicating that these variables are independent.

Regression models were then tested to evaluate the predictive power of independent variables (maternal characteristics such as stress and modulation during shared reading) on dependent variables (child characteristics such as emotional and behavioral difficulties, internalizing and externalizing factors, and socio-emotional development). The aim was to assess whether modulation in maternal interactive style could moderate the effect of maternal stress on child characteristics (SDQ and IDADI), a hypothesis based on findings by Valladares [34] and Shi et al. [33]. No model was constructed with cognitive development (measured by IDADI) as the outcome variable, as it was only related to modulation and not to most of the variables of interest.

Model 1: The first model used the internalizing factor of the SDQ as the dependent variable. The results showed that only maternal stress was a significant predictor of variation in this measure, indicating that maternal behavior during reading interactions does not appear to affect children’s internalizing characteristics. Similarly, modulation did not act as a moderating factor in the impact of stress on children’s difficulties. These findings suggest that internalizing symptoms are associated solely with maternal stress; higher maternal stress is linked to greater internalizing difficulties in children.

Parents and other caregivers experiencing high-stress levels may contribute to the creation of more unpredictable and anxiety-inducing environments [11]. They may resort to more intense or unexpected disciplinary measures [35]. In summary, communication with their children may become impaired, less effective, and less affectionate. A Chilean study [9] found that parental stress significantly predicted encouragement in a parental behavior observation protocol, although it did not find associations with responsiveness and affection. Even so, in contexts of unpredictability, uncertainty, and insecurity, children may exhibit higher levels of anxiety, including internalizing symptoms [36]. Such phenomena could explain the results of this study.

Model 2: The second model focused on the externalizing factor of the emotional and behavioral difficulties scale. Like the previous analysis, maternal stress remained a significant variable in the regression. Modulation during shared reading, considered in isolation, did not contribute significantly to this model. However, its interaction with maternal stress showed a slight contribution to the variation in the externalizing factor, with borderline statistical significance.

Although not entirely conclusive, these data suggest a potential moderating effect of maternal interactive style (specifically, modulation in interaction) on the impacts of maternal stress on children’s emotional and behavioral characteristics. This fact could mean that mothers with higher modulation scores—even those with high stress levels—might have children with fewer externalizing behavioral and emotional difficulties. Conversely, mothers with lower affectionate modulation during shared reading might have children whose externalizing difficulties remain strongly linked to maternal stress levels. In simpler terms, a modulated interactive style could help reduce the relationship between maternal stress and children’s emotional difficulties.

A recent systematic review and meta-analysis [37] evaluated the association between parental stress and externalizing behaviors in childhood and adolescence. The included studies revealed a small but significant effect of prenatal stress on symptoms such as reactive/aggressive behaviors, hyperactivity, and impulsivity. These findings support the present results, providing more robust evidence for the impact of maternal stress on children’s externalizing behavior.

Model 3: The third model aligns with the previous discussion, using the total SDQ score as the dependent variable, encompassing both internalizing and externalizing aspects. This regression model yielded results similar to Model 2: maternal stress as a significant predictor of child difficulties, with its effect slightly moderated by the modulation dimension of maternal interactive style during shared reading. Considering the separate SDQ factors, it appears that externalizing aspects contributed more to the predictive coefficient of the overall model.

Even with borderline statistical significance, the results of these analyses support the hypothesis that a more engaged and affectionate interaction style may mitigate the potential negative effects of adult stress on children. Along these lines, a study conducted in the United States examined the possible impacts of shared reading on parental stress and relational health between parents and children aged 6 to 18 months [16]. The study considered parental affection and sensitivity as variables of interest. The authors aimed to investigate whether shared reading contexts could benefit parents and the parent–child relationship beyond already known outcomes, such as language development and reading skills. Their findings demonstrated that shared reading practices at 6 months of age were associated with longitudinal increases in parental affection and sensitivity, as well as reductions in parental stress levels at 18 months.

A recent longitudinal study by Sasayama et al. [38] found that mother-to-infant bonding significantly mediates the effect of maternal postpartum depression on children’s emotional and behavioral difficulties in sixth grade. These findings resonate with the focus of the present study, highlighting how bonding and high-quality interaction can moderate the impact of maternal psychosocial variables on child characteristics. Conducted with Japanese mother–child dyads, the study also employed the SDQ to assess child outcomes. Structural equation modeling revealed that bonding difficulties mediated 34.6% of the total effect of postpartum depressive symptoms on child difficulties.

Theoretical frameworks and empirical findings, as highlighted by a narrative review conducted by Boissel et al. [39], suggest that positive interaction contexts, such as shared reading, can promote beneficial outcomes in children’s behavioral and emotional aspects. It is promising that even in a study like this one—with a small sample size, video analyses, and hetero-reported scales—such trends emerged, albeit modestly.

Model 4: Finally, the fourth model yielded results similar to the first model. Only maternal stress emerged as a statistically significant predictor. Neither modulation during shared reading nor its interaction with maternal stress was a significant variable in predicting socio-emotional development as measured by the IDADI. It appears plausible that adult stress is related to child developmental characteristics, particularly in the socio-emotional domain [36]. However, the findings of this study do not support the hypothesis that the interactive style during shared reading acts as a potential moderator of this impact.

Additionally, the importance of maternal education in the models studied here cannot be overlooked. In the first three models, which focused on child difficulties, maternal education emerged as a significant and relevant covariate. Notably, this variable consistently had the highest coefficient in all models. Statistically, this indicates that variations in maternal education scores are associated with significant changes in child characteristics scores. Although it is a secondary variable for this study, its importance in the variation of target outcomes cannot be ignored.

This trend is well-documented in child development science [40]. Maternal education often emerges as one of the factors most associated with child measures, whether cognitive, emotional, or social [41]. The consistent appearance of this phenomenon in the present study, aligning closely with previously reported findings in the scientific literature, may serve as an interesting indicator of validity for the methods employed here.

## 5. Conclusions

This study aimed to investigate the predictive relationship between maternal stress and children’s emotional and behavioral characteristics. Additionally, it sought to assess whether maternal interactive style during shared reading could moderate the relationship between maternal stress and child characteristics. Video analyses of dyads engaging in shared reading were conducted alongside applying psychometric instruments to measure the variables of interest.

The results showed that maternal stress was not associated with interactive style during shared reading, potentially indicating that mothers were able to regulate their emotional state effectively when interacting with their children through a children’s book. Furthermore, maternal stress was associated with outcomes in child characteristics, particularly emotional and behavioral difficulties, and the socio-emotional dimension. Among the behavioral categories in shared reading, modulation emerged as the most relevant. Although its role as a moderator in the regression model was only borderline significant, it appeared as a potential moderating variable between maternal stress and child emotional and behavioral characteristics.

Thus, shared reading can be considered a high-quality, protective context for both parents and children, as it helps participants in the interaction regulate their emotions or, at the very least, manage their stress levels. The potential importance of prosodic modulation during reading interactions is also noteworthy, as this strategy could positively impact children’s emotional and behavioral development.

These findings carry relevant implications for early intervention and parent education programs. Given that modulation during shared reading emerged as a potentially protective factor—even if only borderline significant—it may be beneficial to include training on prosodic modulation and expressive reading techniques in caregiver support initiatives. Encouraging parents to engage in emotionally attuned, interactive reading could help foster more positive adult–child relationships and support children’s socio-emotional development, particularly in families experiencing elevated stress. Shared reading, therefore, represents a low-cost, scalable strategy that could be incorporated into public health and educational efforts aimed at promoting healthy development and enhancing parental well-being.

An important limitation of this study is the reliance on a single informant for both maternal stress and child outcomes. This introduces the possibility of shared method variance and perception bias, whereby the same psychological state (e.g., high maternal stress) may influence both the self-report of stress and the evaluation of the child. Consequently, the associations identified in this study may be partially inflated due to common rater effects, rather than reflecting purely objective relationships between variables. Future studies should include reports from multiple informants (e.g., fathers and teachers) or objective assessments to minimize this potential bias.

The limited power to detect interaction effects (1 − β = 0.53) may have contributed to the borderline significance observed. While this is a limitation, the exploratory nature of the study and the complexity of video-coded observational data—common in developmental and clinical psychology—justify a more flexible interpretation. Nonetheless, the findings should be interpreted with caution, and future studies should replicate these results using larger and more diverse samples to enhance statistical sensitivity.

Another important limitation concerns the absence of other psychosocial variables that may be associated with both maternal stress and child development outcomes. Factors such as maternal depression, anxiety, and bonding difficulties were not assessed in the present study, which may act as potential confounders in the observed associations. These variables could influence both the perception and reporting of child behavior, as well as the quality of adult–child interactions. Future research should incorporate a broader set of maternal mental health indicators to better isolate the specific role of stress and deepen the understanding of its unique impact on child outcomes.

Additionally, the influence of shared reading in reducing or mitigating parental stress—and how this may benefit child characteristics—remains a promising topic for further exploration. Specifically, prosodic modulation during reading interactions could serve as a focal point for interventions to promote healthy development and improve the quality of adult–child interactions.

## Figures and Tables

**Table 1:** Descriptive data of target variables in the study (*n* = 91).

	Mean (*SD*)	Minimum Raw Value	Maximum Raw Value	*Skewness* (*SE*)	*Kurtosis* (*SE*)	Cronbach’s Alpha
PSS-14 Total	26.27 (8.14)	8	49	0.40 (0.25)	−0.23 (0.50)	0.85
PSS-14 Resources	11 (4.90)	1	24	0.40 (0.25)	−0.13 (0.50)	0.83
PSS-14 Difficulties	15.27 (4.34)	7	25	0.10 (0.25)	−0.35 (0.50)	0.77
Interactive Style in Shared Reading (SR)						
Teaching	3.88 (3.06)	0	14	1.17 (0.25)	1.24 (0.50)	
Prompting	9.68 (7.18)	0	39	1.55 (0.25)	3.51 (0.50)	
Reinforcement	2.89 (2.41)	0	15	1.57 (0.25)	5.70 (0.50)	
Connection	0.35 (.70)	0	3	2.10 (0.25)	3.92 (0.50)	
Modulation	5.93 (4.59)	0	19	0.88 (0.25)	0.22 (0.50)	
Narration	11.78 (6.64)	0	29	0.35 (0.25)	−0.43 (0.50)	
SDQ Total	13.62 (6.77)	0	34	0.58 (0.25)	0.41 (0.50)	0.80
SDQ Internalizing	5.46 (3.55)	0	17	1.10 (0.25)	1.13 (0.50)	0.70
SDQ Externalizing	8.16 (4.25)	0	18	0.14 (0.25)	−0.49 (0.50)	0.77
IDADI						
Cognitive	81.15 (17.75)	40	119	−0.15 (0.30)	−0.44 (0.60)	
Socio-emotional	93.73 (12.28)	77	129	1.16 (0.27)	1.09 (0.54)	

Legend: *SD* = standard deviation; *SE* = standard error; PSS-14 = Perceived Stress Scale, 14-item version; SR = shared reading; SDQ = Strengths and Difficulties Questionnaire; IDADI = Dimensional Inventory for Child Development Assessment.

**Table 2:** Pearson’s linear correlation analyses between variables of interest (*n* = 91).

	SDQtot	SDQint	SDQext	Cog	SE
PSStot	0.44 ***	0.37 ***	0.41 ***	−0.12	−0.43 ***
PSSrec	0.34 **	0.24 *	0.34 **	−0.14	−0.37 **
PSSdif	0.45 ***	0.41 ***	0.38 ***	−0.06	−0.38 **
ModLC	−0.32 **	−0.30 **	−0.28 **	0.26 *	0.13

Note: only correlations relevant to the study objectives are included. Legend: PSStot = PSS-14 total; PSSrec = PSS-14 resources factor; PSSdif = PSS-14 difficulties factor; ModLC = modulation category in maternal interactive style during shared reading; SDQtot = SDQ total; SDQint = SDQ internalizing factor; SDQext = SDQ externalizing factor; Cog = cognitive dimension of IDADI; SE = socio-emotional dimension of IDADI. * *p* < 0.05; ** *p* < 0.01; *** *p* < 0.001.

**Table 3:** Summary of multivariate regression results for child socioemotional outcomes.

Model	Outcome Variable	Significant Predictors	Interaction Term (Modulation × Stress)	*R* ^2^
1	SDQ—Internalizing	Maternal stress, Maternal education	Not significant (*p* = 0.20)	0.31
2	SDQ—Externalizing	Maternal stress, Maternal education	Borderline (*p* = 0.07)	0.29
3	SDQ—Total	Maternal stress, Maternal education	Borderline (*p* = 0.07)	0.38
4	IDADI—Socio-emotional	Maternal stress	Not significant (*p* = 0.39)	0.19

## Data Availability

Dataset available on request from the authors.
